# Evaluation of Integrin α_v_β_3_ Expression in Murine Xenograft Models: [^68^Ga]Ga-DOTA-C(RGDfK) PET Study with Immunohistochemical Confirmation

**DOI:** 10.3390/diagnostics11071295

**Published:** 2021-07-19

**Authors:** Kosuke Mitsuyuki, Tadashi Watabe, Sadahiro Naka, Yuwei Liu, Mitsuaki Tatsumi, Eku Shimosegawa, Hiroki Kato

**Affiliations:** 1 Department of Nuclear Medicine and Tracer Kinetics, Graduate School of Medicine, Osaka University, Suita 565-0871, Japan; u867869d@ecs.osaka-u.ac.jp (K.M.); liu@tracer.med.osaka-u.ac.jp (Y.L.); kato@tracer.med.osaka-u.ac.jp (H.K.); 2 Department of Radiology, Osaka University Hospital, Suita 565-0871, Japan; naka@tracer.med.osaka-u.ac.jp (S.N.); m-tatsumi@radiol.med.osaka-u.ac.jp (M.T.); 3 Department of Molecular Imaging in Medicine, Graduate School of Medicine, Osaka University, Suita 565-0871, Japan; Eku@mi.med.osaka-u.ac.jp

**Keywords:** [^68^Ga]Ga-DOTA-c(RGDfK), tumor angiogenesis, tumor blood flow, small-animal positron emission tomography, C6 glioma, MIA PaCa-2

## Abstract

Tumor blood flow (TBF) is related to drug delivery and hypoxia, both of which can impact the efficacy of anti-cancer therapies. Although integrin α_v_β_3_ expression is related to tumor angiogenesis, it remains unclear whether the degree of angiogenesis affects TBF. This study aimed to evaluate the expression of integrin α_v_β_3_ in mouse tumor models using [^68^Ga]Ga-DOTA-c(RGDfK) peptide positron emission tomography (PET) and immunohistochemical staining. PET studies were conducted using mouse C6 glioma models and MIA PaCa-2 (*n* = 6 each). The [^68^Ga]Ga-DOTA-c(RGDfK) peptide was injected via the tail vein (2.17 ± 0.28 MBq), and 10 min static PET scans were performed. Immunohistochemical analysis was conducted using an integrin α_V_β_3_ antibody. [^68^Ga]Ga-DOTA-c(RGDfK) peptide PET revealed higher uptake of the radiotracer in C6 gliomas than in MIA PaCa-2 tumors. The mean standardized uptake value was significantly higher in C6 gliomas (0.35 ± 0.058) than in MIA PaCa-2 tumors (0.17 ± 0.045). Histological analysis revealed intense integrin α_V_β_3_ expression in the C6 gliomas, whereas the MIA PaCa-2 tumors had low expression levels. This study showed that the expression of integrin α_v_β_3_ can be differentiated by the [^68^Ga]Ga-DOTA-c(RGDfK) peptide, suggesting the potential applicability of this peptide in the evaluation of the relationship between angiogenesis and TBF.

## 1. Introduction

Angiogenesis is an important factor that contributes to invasive tumor growth and metastasis and is related to cancer progression [1,2,3]. Anti-angiogenic therapy is widely used in clinical practice to normalize the formation of tumor vessels in several types of cancer [4,5,6,7]. Although the mechanisms are not completely understood, the upregulation of integrin α_v_β_3_ is observed in activated vascular endothelial cells and solid tumor cells, and plays an important role in tumor angiogenesis and metastasis [8,9,10]. Non-invasive imaging using positron emission tomography (PET) is an important tool for understanding tumor blood flow (TBF) and the tumor microenvironment, as it facilitates the clinical translation of novel therapies and provides essential information for selecting treatment strategies in clinical settings. To visualize and quantify the expression levels of integrin α_v_β_3_, [^68^Ga]Ga-labeled Arg-Gly-Asp (RGD) peptides have been used due to their high affinity and selectivity for integrin α_v_β_3_ [11,12]. As integrin α_v_β_3_ expression has been established as a surrogate marker of angiogenic activity, PET imaging using radiolabeled RGD peptides could potentially be used as an early indicator of the efficacy of anti-angiogenic therapies [11,12].

Previous studies have reported that TBF is related to drug delivery and the degree of hypoxia, both of which impact the efficacy of anti-cancer therapies [13,14]. We have recently shown that a decrease in TBF, detected using [^15^O]water PET following bevacizumab treatment, was positively correlated with longer progression-free survival in patients with non-small cell lung cancer [15]. However, it is still unclear whether the degree of angiogenesis affects TBF. In this preliminary study, we aimed to determine whether the expression level of integrin α_v_β_3_ can be used to differentiate two tumor cell xenograft models in mice using [^68^Ga]Ga-1,4,7,10-tetraazacyclododecane-1,4,7,10-tetraacetic acid-cyclo-(Arg-Gly-Asp-D-Phe-Lys) {[^68^Ga]Ga-DOTA-c(RGDfK)} peptide PET and immunohistochemical staining.

## 2. Materials and Methods

### 2.1. Synthesis of the [^68^Ga]Ga-DOTA-c(RGDfK) Peptide

[^68^Ga]Ga^3+^ was eluted as [^68^Ga]GaCl_3_ using a ^68^Ge/^68^Ga generator (ITG Isotopes Technology GmbH, Garching, Germany) and 4 mL of 0.05 M HCl. Subsequently, 1 mL of [^68^Ga]GaCl_3_ and 50 µL of 2 M sodium acetate were added to 20 µg (1 mg/mL in water) of DOTA-c(RGDfK) acetate (ABX, Radeberg, Germany) and gently mixed. [^68^Ga]Ga-DOTA-c(RGDfK) was obtained by allowing the mixture to react at 95 °C for 10 min, which was administered to mice without purification after measuring the pH value using a pH meter (HORIBA, Kyoto, Japan) with the ultraviolet detector set to wavelength 220 nm and determining the radiochemical purity. Radiochemical purity measurement was performed with a Shimadzu HPLC Prominence system (SHIMADZU, Kyoto, Japan) and Gabi-star radioactivity detector (Elysia-Raytest, Straubenhardt, Germany) using kinetex EVO C18 (250 × 4.6 mm) column (Phenomenex, Torrance, CA, USA). The eluent used for gradient analysis was acetonitrile (solvent A) and 0.1% trifluoroacetic acid (solvent B) (A: 0% from 0 to 1.5 min, 0% to 30% from 1.5 to 18 min, 30% to 60% from 18 to 21 min, and then 60% from 21 to 24 min), and the flow rate was set to 1.0 mL/min.

### 2.2. Animal Model Preparation

Male BALB/cSlc-*nu* mice (5 weeks old) were obtained from Japan SLC, Inc. (Hamamatsu, Japan). C6 glioma cells, which were obtained from a rat cell line derived from glial cell tumors induced by N-Nitroso-N-methylurea, were purchased from RIKEN

BioResource Research Center (Tsukuba 305-0074, Japan). MIA PaCa-2 cells derived from a human pancreatic cancer cell line were obtained from the American Type Culture Collection (Manassas, VA, USA). The C6 glioma cells were cultured in Minimum Essential Medium (MEM) (Sigma-Aldrich Japan, Tokyo, Japan), whereas the MIA PaCa-2 cells were cultured in Dulbecco’s Modified Eagle Medium (Sigma-Aldrich Japan, Tokyo, Japan) containing 10% fetal bovine serum (Sigma-Aldrich Japan, Tokyo, Japan) and a penicillin/streptomycin/amphotericin B mixture. All cells were placed in an incubator filled with a gas mixture of 5% CO_2_ and 95% air at 37 °C for 3 weeks. To generate the tumor xenograft models, the cultured cells were detached from the dishes using 0.25% (*w*/*v*) trypsin. The 5-week-old BALB/cSlc-*nu* mice received subcutaneous injections of either a 0.2 mL mixture of C6 glioma cells (1.0 × 10^7^ cells) and Matrigel or a 0.2 mL mixture of MIA PaCa-2 cells (1.0 × 10^7^ cells) and Matrigel in the left shoulder. The tumor xenograft mice implanted with C6 glioma cells (*n* = 6) had a body weight of 24.55 ± 0.91 g, and those implanted with MIA PaCa-2 cells (*n* = 6) had a body weight of 24.42 ± 1.51 g. The tumor size of the C6 glioma was 272.7 ± 104.7 mm^3^ and that of the MIA PaCa-2 was 759.1 ± 327.7 mm^3^, as determined by PET.

### 2.3. PET and Image Analysis

PET/computed tomography (PET/CT) was performed 3 weeks after tumor implantation. In both models, the [^68^Ga]Ga-DOTA-c(RGDfK) peptide was injected via the tail vein of the mice (dose in C6 glioma model: 2.18 ± 0.34 MBq; dose in MIA PaCa-2 model: 2.17 ± 0.25 MBq) under isoflurane anesthesia. A micro-PET/CT scanner (Inveon, Siemens, Munich, Germany) was used for small-animal PET scanning. The model mice were subjected to 70 min dynamic PET scans (C6 glioma model: *n* = 2; MIA PaCa-2 model: *n* = 1). In addition, 10-min static PET scans were performed 60 min after injection in the C6 glioma (*n* = 4) and MIA PaCa-2 (*n* = 5) model mice.

### 2.4. Biodistribution of the Radiotracer

Following PET imaging, all mice were euthanized using an inhalation anesthetic. Some mice (*n* = 3 for the C6 glioma model and *n* = 3 for the MIA PaCa-2 model) were dissected, and the following organs were collected: salivary glands, lungs, heart, spleen, pancreas, kidneys, bone, testes, blood, brain, liver, stomach, small intestine, large intestine, and tumors. Radioactivity and organ weight were measured using a well scintillation counter (BeWell, Molecular Imaging Labo, Osaka, Japan). The percentage accumulation of the [^68^Ga]Ga-DOTA-c(RGDfK) peptide was determined in relation to the injected dose (%ID), and the ratio of the %ID to the weight of each organ (%ID/g) was calculated.

### 2.5. Data Analysis

The concentration of the [^68^Ga]Ga-DOTA-c(RGDfK) peptide was estimated by measuring the standardized uptake value (SUV), corrected based on the injected dose (in MBq) and the animal’s body weight (g). Spherical volumes of interest were placed on the tumor locations in the PET images using PMOD software (Ver 4.0) while referring to the CT images. The mean and maximum SUVs (SUV_mean_ and SUV_max_, respectively) of the [^68^Ga]Ga-DOTA-c(RGDfK) peptide were compared between the C6 glioma (*n* = 6) and MIA PaCa-2 (*n* = 6) xenograft models.

### 2.6. Immunohistochemical Labeling

The tumors were fixed with Mildform 10 N (FUJIFILM Wako Pure Chemical Corporation, Osaka, Japan) overnight. After cryoprotection in 30% sucrose, the frozen tissue slices of the tumors were sectioned (10 µm) using a cryostat and stained for immunohistochemical evaluation using an integrin α_v_β_3_ polyclonal primary antibody (rabbit immunoglobulin G) (bs-1310R, Bioss Antibodies, Woburn, MA, USA) and an anti-rabbit horseradish peroxidase (HRP)-conjugated secondary antibody (Envision + system-HRP labeled polymer; K4003, Dako, Glostrup, Denmark). Following immunohistochemical labeling, the tissue slices were examined under a light microscope (BZ-9000, Keyence, Osaka, Japan).

### 2.7. Statistical Analysis

Unpaired *t*-tests were used to compare the values between the two groups using Excel software (Ver 16.16.27, Microsoft, Redmond, WA, USA). Statistical significance was set at *p* < 0.05.

## 3. Results

[^68^Ga]Ga-DOTA-c(RGDfK) was found to have radioactivity of 46 ± 12 MBq, a pH value of 5, and radiochemical purity of >98%.

**Figure 1:**
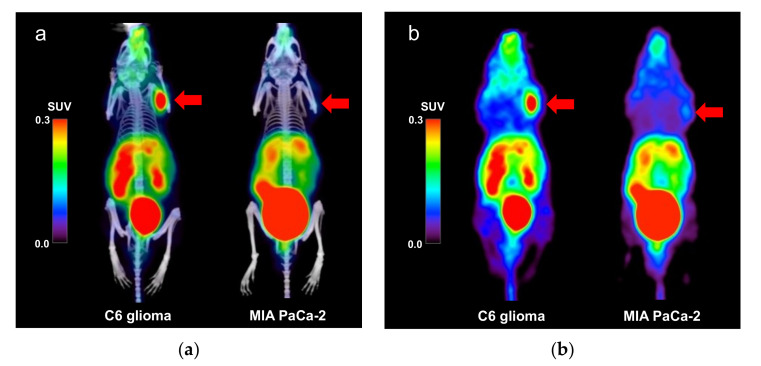
(**a**) Positron emission tomography (PET)/computed tomography (CT) and (**b**) PET images (maximum intensity projection) showing the uptake of the [^68^Ga]Ga-DOTA-c(RGDfK) peptide in the C6 glioma and MIA PaCa-2 model mice. The arrows indicate the locations of the tumor xenografts. Abbreviations: DOTA, 1,4,7,10-tetraazacyclododecane-1,4,7,10-tetraacetic acid; RGD, Arg-Gly-Asp.

Sample PET/CT images showing uptake of the [^68^Ga]Ga-DOTA-c(RGDfK) peptide in the C6 glioma and MIA PaCa-2 model mice at 60 min post tracer injection are shown in Figure 1. [^68^Ga]Ga-DOTA-c(RGDfK) peptide PET revealed high uptake in the C6 glioma xenografts, whereas mild uptake was observed in the MIA PaCa-2 xenografts.

**Figure 2:**
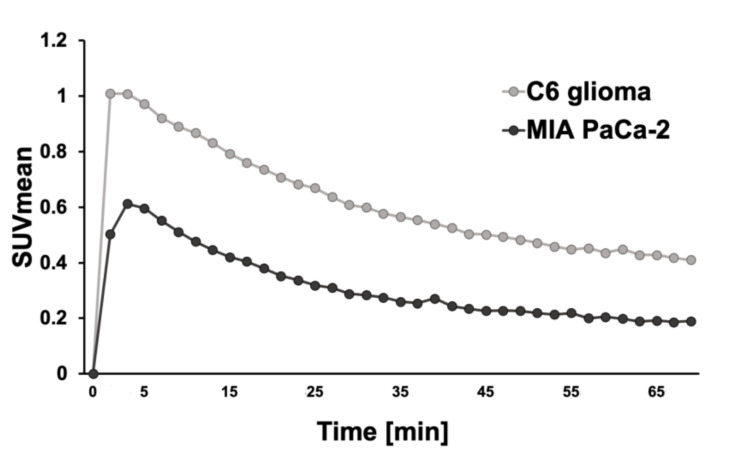
The time-activity curves of the mean standardized uptake value (SUV_mean_) of the C6 glioma mouse model (*n* = 2) and MIA PaCa-2 (*n* = 1).

Time-activity curves of the dynamic PET scans for the C6 glioma mouse model (*n* = 2) and the MIA PaCa-2 mouse model (*n* = 1) for the 70 min period following the injection are shown in Figure 2.

**Figure 3:**
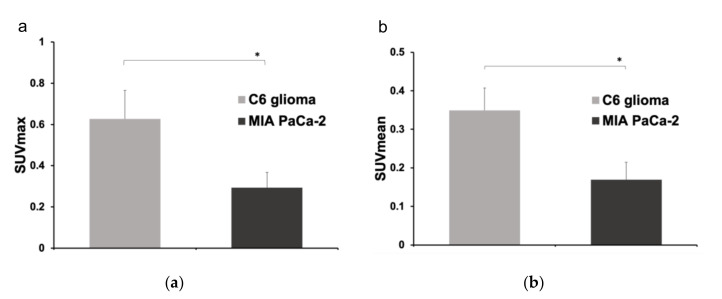
Comparison of (**a**) the maximum standardized uptake value (SUV_max_) and (**b**) the SUV_mean_ between the C6 glioma (*n* = 6) and MIA PaCa-2 (*n* = 6) mouse models (*: *p* < 0.05 as determine by an unpaired *t*-test).

Uptake of the [^68^Ga]Ga-DOTA-c(RGDfK) peptide was significantly higher in the C6 glioma model mice than in the MIA PaCa-2 mice (Figure 3). The SUV_max_ and SUV_mean_ were 0.63 ± 0.14 and 0.35 ± 0.06 in the C6 glioma mice, respectively, both of which were significantly higher than the SUV_max_ and SUV_mean_ of 0.29 ± 0.07 and 0.17 ± 0.45, respectively, in the MIA PaCa-2 mice (*p* < 0.05 for both). The results of the semi-quantitative analysis were consistent with those of immunohistochemical staining.

**Figure 4:**
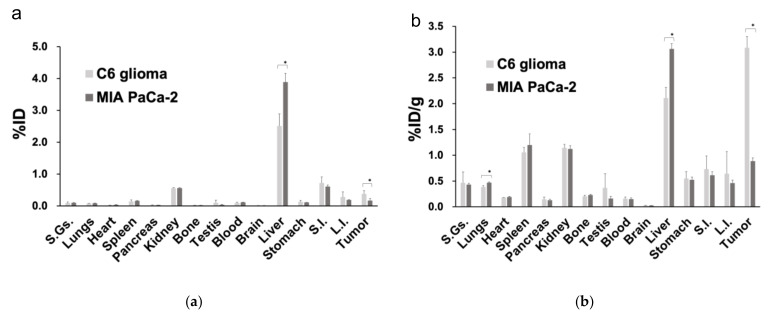
Biodistribution analysis comparing the percentage accumulation of the [^68^Ga]Ga-DOTA-c(RGDfK) peptide based on (**a**) the injected dose and (**b**) the ratio of the injected dose to the weight of each organ (*: *p* < 0.05 as determined by an unpaired *t*-test). Abbreviations: %ID, percentage of the injected dose; %ID/g, ratio of the percentage of the injected dose over the weight of each organ; S.Gs.: salivary glands; S.I.: small intestine; L.I.: large intestine.

The results of the biodistribution analysis are shown in Figure 4. The %ID and %ID/g values of the tumor tissue were 0.40 ± 0.10 and 3.1 ± 0.20, respectively, in the C6 glioma model, both of which were significantly higher than the %ID and %ID/g values of 0.20 ± 0.10 and 0.90 ± 0.10, respectively, in the MIA PaCa-2 model (*p* < 0.05 for both). For organs other than the liver, comparable accumulation was observed in both the C6 glioma and MIA PaCa-2 xenografts.

**Figure 5:**
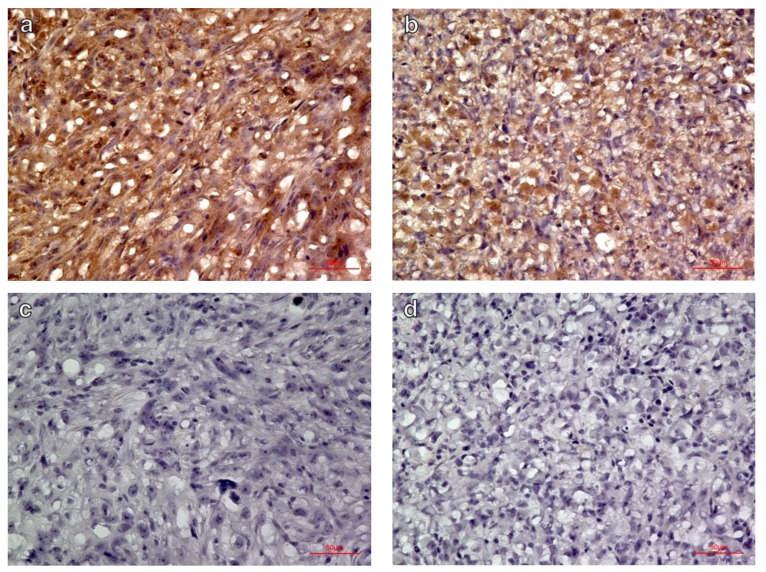
Immunohistochemical labeling of integrin α_v_β_3_ in (**a**) C6 glioma, (**b**) MIA PaCa-2 xenografts, and negative controls of (**c**) C6 glioma and (**d**) MIA PaCa-2 without primary antibody (magnification ×400). The C6 glioma showed more intense expression of integrin α_V_β_3_ than did the MIA PaCa-2.

Qualitative immunohistochemical evaluation revealed intense integrin α_V_β_3_ expression in the C6 glioma model (*n* = 6), but lower expression in the MIA PaCa-2 model (*n* = 6) based on the relatively fainter labeling (Figure 5).

## 4. Discussion

In the present study, PET imaging revealed higher accumulation of the [^68^Ga]Ga-DOTA-c(RGDfK) peptide in the C6 glioma model than in the MIA PaCa-2 model, which was consistent with the high expression of integrin α_v_β_3_ observed in the immunohistochemical staining experiment. These results coincided with the result of the biodistribution analysis.

This was also consistent with the findings of our previous study in which we performed PET using a different radiotracer, [^15^O]water, to show that C6 glioma xenografts exhibited higher TBF than MIA PaCa-2 xenografts using the same models [16]. [^68^Ga]Ga-DOTA-c(RGDfK) peptides have high affinity and selectivity for integrin α_v_β_3_, the expression of which is related to angiogenesis and may reflect the TBF in C6 glioma and MIA PaCa-2 xenograft models [11].

Biodistribution analysis revealed significantly higher accumulation of [^68^Ga]Ga-DOTA-c(RGDfK) in the C6 glioma than in that of the MIA PaCa-2 in both %ID and %ID/g. The accumulation was also higher in the liver of the MIA PaCa-2 than in that of the C6 glioma in both %ID and %ID/g. In the evaluation of the time-activity curve of the SUV_mean_ in the liver, the SUV_mean_ of the MIA PaCa-2 was constantly higher than that of the C6 glioma (data not shown). The reason for these differences in the liver remains unknown.

Vascularized tumors induce angiogenesis in host microvessels, have the potential to expand the population of cells they are composed of rapidly, and have a propensity to metastasize. However, although angiogenesis is necessary, it alone is insufficient to induce tumor growth and metastasis [17,18]. It has also been suggested that angiogenesis depends on the adhesive interactions of vascular cells, and the adhesion receptor integrin α_v_β_3_ has been identified as a marker of angiogenesis in vascular tissue [19,20]. Yoshimoto et al. demonstrated that angiogenic blood vessels overexpress integrin α_v_β_3_, similar to the overexpression that occurs in neovascularized tumors [21]. Limited integrin α_v_β_3_ expression has been observed in other microvascular beds and organs, suggesting that integrin α_v_β_3_ could be a suitable target for active tumor imaging [21].

Although integrin α_v_β_3_ is known to be an important factor in tumor angiogenesis, its exact functional role is not clearly understood. We previously reported that C6 glioma xenografts had higher TBF than did MIA PaCa-2 xenografts, and there was a relationship between the difference in TBF and vascular endothelial growth factor (VEGF) expression levels, as well as microvessel density [16]. VEGF expression is also reported to be associated with microvessel density [22], and it is a key mediator of angiogenesis in cancer [23,24,25,26]. Collectively, these findings suggest a possible correlation between TBF and tumor angiogenesis. In the present study, we observed higher expression of integrin α_v_β_3_ in C6 gliomas than in MIA PaCa-2 xenografts; this is consistent with the results of our previous study, which reported a higher TBF in a C6 glioma model. Although further evaluation is necessary, the expression of integrin α_v_β_3_, which is related to angiogenesis, may reflect TBF.

The applications of [^68^Ga]Ga-DOTA-c(RGDfK) have been widely recognized. Animal studies and clinical studies have been conducted for the angiogenesis imaging of the tumors, such as breast cancer, glioma, and lung cancer, using the ^68^Ga-labeled peptides targeting integrin α_v_β_3_, including NOTA-RGD-peptides and DOTA-RGD-peptides [27]. The efficacy of bevacizumab for non-small cell lung cancer has been evaluated using [^15^O]water PET, which was superior to CT for assessing the therapeutic effect of treatment at an early stage [15]. We speculated that if angiogenesis reflected the TBF, the therapeutic effect of treatment could be evaluated using [^68^Ga]Ga-DOTA-c(RGDfK) peptide PET. Another study reported that [^68^Ga]Ga-1,4,7-triazacyclononane,1-glutaric acid-4,7-acetic acid-c(RGDfK) PET {[^68^Ga]Ga-NODAGA-c(RGDfK) PET} was useful for in vivo imaging of angiogenesis and could be useful for monitoring the effects of anti-angiogenic treatment; for example, with or without bevacizumab and/or temozolomide chemotherapy [28]. In addition, bevacizumab targets VEGF and inhibits angiogenesis [26,29,30,31]. Therefore, the results of the present study suggest that [^68^Ga]Ga-DOTA-c(RGDfK) peptide PET could be useful for evaluating angiogenesis and may facilitate treatment strategies for targeting VEGF in the future.

This study has some limitations. First, the number of xenograft model animals was limited (*n* = 12), and future experiments using such models should have larger sample sizes. Second, we only used the C6 glioma and MIA PaCa-2 tumor models; therefore, future studies should assess the generalizability and further evaluate the utility of [^68^Ga]Ga-DOTA-c(RGDfK) peptide PET with the use of other tumor models. Third, since this research was based on our previous study in which we investigated TBF in C6 glioma and MIA PaCa-2 models, we did not evaluate the direct relationship between the degree of angiogenesis and TBF due to the limited availability of ^68^Ga and the technical difficulty to receive a supply of [^15^O]water, a short half-life radionuclide. Although major differences between nude rats and nude mice used to evaluate the characteristics of the xenografts are not expected, a direct comparison to angiogenesis and TBF should be performed in the next study using the same xenograft model and in the same animals. In addition, relative changes in angiogenesis and TBF in response to anti-angiogenic therapy should be further evaluated in future studies to identify better cancer treatment strategies.

## 5. Conclusions

Both [^68^Ga]Ga-DOTA-c(RGDfK) peptide PET and immunohistochemical staining revealed that the C6 glioma xenografts exhibited a higher expression of integrin α_V_β_3_ than did the MIA PaCa-2 xenografts. This imaging technique could be applicable in the evaluation of tumor angiogenesis and the detection of precise tumor phenotyping, which may lead to more effective treatment strategies in the future.

## Data Availability

Data presented in this study may be available through communication with the corresponding author.
